# Gender and Nightshift Work: A Cross Sectional Study on Sleep Quality and Daytime Somnolence

**DOI:** 10.3390/brainsci13040607

**Published:** 2023-04-03

**Authors:** Rosamaria Lecca, Michela Figorilli, Elisa Casaglia, Carla Cucca, Federico Meloni, Roberto Loscerbo, Sara De Matteis, Pierluigi Cocco, Monica Puligheddu

**Affiliations:** 1 Interdepartmental Sleep Research Centre, University of Cagliari, 09042 Cagliari, Italy; 2 Department of Medical Sciences and Public Health, University of Cagliari, 09042 Cagliari, Italy; 3 Centre for Occupational and Environmental Health, Division of Population Health, University of Manchester, Manchester M13 9PT, UK

**Keywords:** nightshift work, female gender, Epworth Sleepiness Scale, Pittsburgh Sleep Quality Index, shift work tolerance

## Abstract

A few studies suggested that female nightshift workers suffer more frequently from sleep deprivation and insomnia. We conducted a cross-sectional survey in two different occupational settings to address gender-related differences in nightshift work adaptation. We used the Epworth Sleepiness Scale and the Pittsburgh Sleep Quality Index questionnaires to quantify daytime sleepiness and sleep quality among 156 workers, 91 from a ceramic tile factory and 65 healthcare workers, including hospital doctors, nurses, and nurse assistants. Seventy-three percent of participants (40 women and 74 men) were engaged in nightshift work. We used logistic regression analysis to predict daytime sleepiness and poor sleep quality as a function of personal and lifestyle variables and nightshift work. The female gender showed a strong association with both daytime sleepiness and poor sleep quality. Results were also suggestive of an increase in the risk of daytime sleepiness associated with nightshift work and being married. Our results confirm that women are especially vulnerable to sleep disruption. Promoting adaptation to nightshift work requires special attention towards gender issues.

## 1. Introduction

Shift work is essential to sustaining the requirements of our 24 h society. However, working at unusual times causes the misalignment of the sleep–wake cycle with the endogenous circadian rhythm, resulting in acute and long-term adverse health outcomes.

The consequences are sleep loss, sleep disruption, and daytime somnolence [1], which can be limited to the days immediately following the nightshifts or can become long-lasting consequences, leading to a specific sleep disorder, called Shift Work Disorder (SWD) [2], a circadian rhythm disorder characterized by insomnia and excessive daytime sleepiness [3].

Poor alertness during the night shift and daytime sleepiness may impair cognitive performance, increasing the risk of workplace injuries and errors at work and car accidents while commuting back home after the night shift [4].

In the long run, sleep deprivation and circadian disruption lead to higher stress levels and metabolic changes that can have long-term health impacts [5], such as an increased incidence of metabolic syndrome, type II diabetes [6], cardiovascular diseases, and stroke [7]. Besides, the International Agency for Research on Cancer (IARC) classifies nightshift work as a probable human carcinogen (Group 2A), with limited epidemiological evidence for an association with breast cancer, prostate cancer, and colorectal cancer [8].

Especially among female nightshift workers, the disruption of the circadian rhythm due to exposure to light at night has been linked to an elevated risk of breast cancer. However, the nature of the association with shift work, whether direct or due to confounding, has not been established yet [9].

Furthermore, female shift workers may experiment menstrual cycle disruption and may be at risk for lower fertility or even harmful condition during pregnancy, such as gestational diabetes and hypertension [10].

Shift work tolerance was defined in 1979 as a level of adaptation to work shifts high enough to prevent suffering from adverse health consequences [11]. Two main factors, namely well-being in terms of sleep quality, mental health and quality of life, and physical health, affect shift work tolerance. Questionnaires on insomnia, circadian rhythm, anxiety, and depression, and others on pain and perception of poor quality of life, can effectively explore either factor [12].

Several other characteristics, such as age, gender, chronotype, marital status, having children, job satisfaction, and individual coping mechanisms, influence the ability to adapt to shift work [13]. Moreover, obesity reportedly prevails among nightshift workers because of the associated unhealthy eating habits [14], and there is some evidence that alcohol consumption [15] and smoking [16] may be detrimental to sleep quality.

A recent consensus document of the Working Time Society recommended addressing research on the role of social, familial, and physiological gender-related differences in shift work adaptation [17].

Robust epidemiological evidence indicates that women suffer from insomnia [18,19,20] and complain about sleep disturbance [19,21] more frequently than men. Recently, a systematic review conducted by Brito et al. confirmed the higher prevalence of insomnia among female shift workers [22].

However, objective investigations have shown a different picture: for instance, an actigraphy study showed better sleep quality, longer sleep duration, and shorter sleep latency among women [23], while polysomnography studies did not support substantial gender-related differences in sleep outcomes [24,25]. Understanding the biological and social factors underlying the gender-related differences in the adaptation to nightshift work effects is crucial for implementing interventional strategies to reduce the health impact of shift work.

Within a preliminary feasibility evaluation of a large project on shift work, while testing the questionnaire, we explored the personal and lifestyle factors that plausibly affect the adaptation to nightshift work in two different occupational settings. We were especially interested in exploring gender-related differences to better address possible interventions to foster tolerance and improve well-being among night shift workers.

## 2. Materials and Methods

We conducted a cross-sectional study on 91 workers from a ceramic tile factory (95% of the total workforce) and a sample of 65 hospital staff (1% of the total personnel employed at the hospital), who attended the Occupational Health outpatient clinic of the Cagliari University Hospital (Sardinia, Italy) for the annual workplace health surveillance between June and July 2018. All subjects were eligible for the study and signed an informed consent form prior to participation. There were no refusals. Sixty-three (10 females and 53 males, 69%) out of the 91 workers from the ceramic tile factory were engaged in a regular, forward rotating shift work scheme: two mornings, two postmeridian shifts, two nights, and three days of rest (MMEENNRRR). The rest of the workforce, consisting of four women and 24 men, had a fixed 8-hour-5-days-per-week daytime work schedule.

Fifty-one (30 females and 21 males, 78%) of the 65 health care staff were engaged in shift work according to the following regular shift rotating schedules: two-morning shifts (M), two postmeridian shifts (E), one-night shift (N), and two rest days (R) (MMEENRR). However, to cover the staff shortage in the holiday seasons, the August and December rotation included two consecutive nights before the rest days (MMEENNRR). The other 14 hospital employees, 13 of whom were females, only worked fixed daytime shifts 5-days a week.

According to the Italian legislation decree N. 66/2003, the definition of nightshift worker indicates one who works not less than 3 h of his/her daily working hours, between midnight and 5am, for at least 80 nights/year. Workers reaching such threshold belong to a category of health-damaging jobs entitled to benefits, such as an earlier retirement and a 15% wage increase. Ceramic tile shift workers worked on average 74 nightshifts/year, and shift working nurses had on average 60 nightshifts/year, therefore not reaching the threshold for the legal definition of engagement in a health-damaging job.

All subjects self-administered the questionnaire while in the waiting room of the Occupational Health outpatient clinic. The questionnaire did not include personal identification data and was composed of two parts: the first part included sociodemographic data, lifestyle habits, sleeping habits, and information about the work schedule, such as the number of night shifts/month, working hours, and type of rota. The second part included three questionnaires, validated in numerous studies and freely available on the Internet: the Epworth Sleepiness Scale (ESS) for the evaluation of somnolence, the Pittsburg Sleep Quality Index (PSQI) for the assessment of quality of sleep, and the Morningness Eveningness Questionnaire (MEQ) for the chronotype definition [26,27,28]. The ESS is composed of 8 items that evaluate the propensity to fall asleep in different circumstances of daily life in a scale from 0 to 3. A score below 11 is considered normal; higher scores are suggestive of daytime somnolence of progressively increasing severity [26]. The PSQI measures sleep quality in adults; it includes a self-assessment scale consisting of seven components that combine 18 questions. Based on the answers, each component is associated with a score ranging 0–3. The sum of scores is binary classified as poor sleep quality if ≥5 or good sleep quality if <5 [27]. Both the ESS and the PSQI questionnaires have profitably been used in assessing shift work tolerance in occupational health settings [29]. The MEQ includes 19 questions about the regular hours of going to bed and waking up, the preferred time of the day for physical and mental activities, and the individual perception of being alert during the day [28]. The score varies by item, and the total score ranges 16–86: a score ≤41 defines a serotine chronotype; a score between 42–58 defines an intermediate chronotype; and a score ≥59 defines a matutine chronotype. Sleeping hours were categorized as regular if 7 or more, and insufficient if less than 7.

### Statistical Methods

We used parametric or non-parametric measures of central tendency and spread as appropriate to describe the study variables, the Student’s t test to compare normally distributed parametric variables by study groups, the Mann–Whitney test to compare ESS and PSQI scores by smoking habit (current smokers vs. never and ex-smokers), alcohol intake (daily, occasional, abstinent), marital status (married vs. single and divorced), chronotype, and nightshift work. We tested categorical variables by study group with the Pearson’s *χ*^2^ test. We also conducted univariate Spearman correlation analyses to explore the relationship between ESS and PSQI scores with age and body mass index (BMI). In all instances, we rejected the null hypothesis when the chance probability associated with the null hypothesis was less than 5%.

For the multivariate analysis, we dichotomized the ESS score (≥11 vs. ≤10) and the PSQI score (≥5 vs. ≤4) as the dependent outcomes. We used unconditional logistic regression models to predict high ESS or PSQI scores adjusting by age, gender, marital status, night shift work, hours of sleep (regular ≥ 7 h/night, sleep-deprived < 7 h/night), and the chronotype. We used the Log-likelihood test to calculate the goodness-of-fit of the regression model [30] and the area-under-the ROC curve to evaluate the model quality [31]. Other covariates, such as the number of children, smoking, alcohol intake, and workplace, did not show an association in the univariate analysis nor improved the models’ goodness-of-fit. The odds ratio (OR) and its 95% confidence interval measured the association of the independent covariates with either outcome. The analysis was conducted with SPSSv20^®^.

## 3. Results

### 3.1. Univariate Analysis of Demographic, and Lifestyle Variables

Workers from the two workplaces differed for several aspects (Table 1). The mean age of study participants was 42.4 years (standard deviation [*sd*] 9.9), and it did not vary by gender (*p* = 0.54); however, on average, the male hospital staff were younger than the female colleagues (*p* = 0.02). BMI was 24.5 on average (*sd* 4.02), and it was higher among men than women (men: 25.4, *sd* 3.72; women: 23.0, *sd* 4.17, *p* < 0.001). Marital status did not vary substantially by gender, but it did differ by the workplace (*p* < 0.001), as having minor children did (*p* < 0.001). A daily alcohol intake was reported by 1/57 (5.3%) female participants vs. 37/99 (37%) men, and they were all from the ceramic tile factory, whilst three-quarters male and half female hospital staff and one-fourth male and one-third female factory workers reported an occasional drinking habit. Current smokers were more prevalent among men (31/99, 31% vs. 7/57, 12%; *p* = 0.008), almost equally by workplace.

Table 2 shows the comparison of individual and lifestyle factors by engagement in nightshift work. In the overall study population, the mean age was similar between nightshift and daytime workers; however, hospital staff working nightshifts were younger than those working daytime only (*p* = 0.001). Additionally, in the general population, BMI was not elevated among nightshift workers, but it was so among nightshift workers from the tile factory (*p* = 0.011). The hospital staff working nightshifts used to be more frequently current smokers and alcohol drinkers, but it was the opposite among factory workers so that over the total study population there was no difference by nightshift work engagement. The prevalence of sleep-deprived subjects was not elevated in nightshift workers in respect to daytime workers.

### 3.2. Univariate Analysis of Daytime Sleepiness, Sleep Quality, and Chronotype

The distribution of chronotypes across the study population was suggestive for a prevalence of the matutine chronotype among daytime factory workers (*p* = 0.046), but not among daytime hospital workers nor in the overall study population. Females more frequently reported sleeping less than 7 h per night (*p =* 0.035). On the other hand, the proportion of sleep deprived workers did not vary by workplace (females *p* = 0.549, males *p* = 0.487) Almost three-quarter of study participants worked nightshifts; the prevalence of nightshift work did not vary by gender nor by workplace. Noteworthy, almost all the male hospital staff (21/22) worked nightshifts vs. 70% of the female staff (*p* = 0.018). Daytime sleepiness, as detected through an ESS score ≥ 11, and sleep quality, as detected by a PSQI ≥ 5, did not vary by workplace (*p* = 0.528, and *p* = 0.819, respectively).

In this study population, sleep deprivation was common among daytime workers, who slept on average 6.1 h (*sd* 1.37). Hours of sleep were significantly less among female daytime workers than the males (5.5 h *sd* 1.19 vs. 6.6 h *sd* 1.11; *t* = 2.89, *p* = 0.005). Such circumstance might have prevented nightshift work to emerge as a condition inducing sleep loss: in fact, in our study, nightshift workers used to sleep on average 6.5 h (*sd* 1.24). Hours of sleep were substantially similar by gender among nightshift workers (*t* = 0.55, *p* = 0.604).

**Figure 1:**
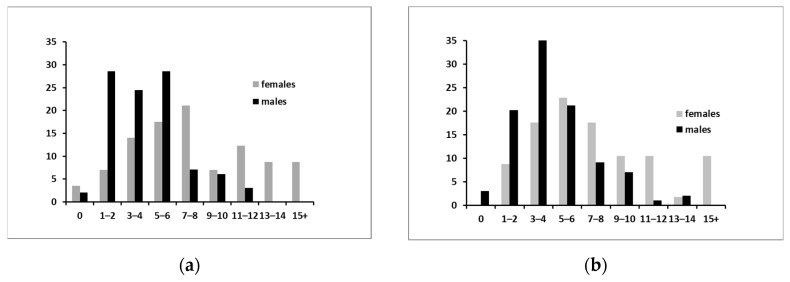
Distribution of the ESS score (**a**) and the PSQI score (**b**) by gender.

In the overall study population, the ESS score distribution among women was shifted towards higher values (Figure 1a), and the median was higher (median = 7, interquartile range (IQ) = 5–11) in respect to men (median = 4, IQ range = 3–8) (Mann–Whitney test = 5.79; *p* < 0.001); thirteen women and three men had an ESS score equal to or above 11 (*p* < 0.001).

Female participants also experienced a poorer sleep quality than men (Mann–Whitney test = 4.60; *p* < 0.001); seventy-four percent of women reported a PSQI score ≥5, suggestive of poor sleep quality, compared to 40% of men (Figure 1b).

The prevalence of ESS scores ≥11 and PSQI scores ≥5 was slightly higher among female nightshift workers than those working daytime. Among males, only three subjects had an ESS score ≥11, suggestive of daytime somnolence: all worked nightshifts. On the other hand, there was no difference in the prevalence of elevated PSQI scores between male nightshift workers and daytime workers.

**Figure 2:**
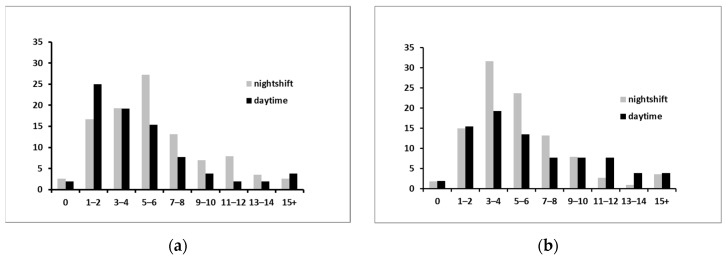
Distribution of the ESS score (**a**) and the PSQI score (**b**) in nightshift workers and daytime workers.

The ESS score and the PSQI score did not vary by working night shifts or fixed daytime shifts (Figure 2a,b) (Mann–Whitney test: ESS = 1.63; *p* = 0.106; PSQI = −0.19; *p* = 0.392). Carrying a morning, an evening, or an intermediate chronotype did not make a difference in the prevalence of daytime somnolence, over the total population, and among night shift workers (not shown in the Tables). However, the matutine chronotype was apparently less frequently represented among nightshift workers (males: *χ*^2^ = 1.83, *p* = 0.176; females: *χ*^2^ = 0.39, *p* = 0.534; total: *χ*^2^ = 2.18, *p* = 0.140).

In the univariate analysis, the ESS and the PSQI scores were moderately correlated in men (Spearman’s correlation coefficient = 0.231, *p* = 0.022) and a very strongly correlated in women (Spearman’s correlation coefficient = 0.906, *p* < 0.0001). Neither the ESS score nor the PSQI score showed a direct correlation with number of monthly night shifts in both genders (male nightshift workers: ESS = −0.204, *p* = 0.081; PSQI = −0.094, *p* = 0.426. female nightshift workers: ESS = 0.025, *p* = 0.833; PSQI = 0.022, *p* = 0.852). BMI and age did not affect the ESS and PSQI scores in both genders. Neither did ESS and PSQI scores vary by marital status, having children, smoking, or alcohol intake (not shown in the Tables).

### 3.3. Multivariate Analysis of the Determinants of Daytime Sleepiness and Poor Sleep Quality

Table 3 shows the results of the logistic regression models predicting ESS scores ≥11, and PSQI scores ≥5. Risk estimates were quite imprecise, due to the small numbers in some cells. Female gender was the strongest risk factor for daytime sleepiness (OR = 15.9, 95% CI 4.20–60.0) and poor sleep quality (OR = 3.91, 95% CI 1.75–8.73). The statistical power was not sufficient to exclude chance as responsible of the elevated risk of daytime sleepiness associated with being married (OR = 2.62, 95% CI 0.78–8.83) and working nightshifts (OR = 1.49, 95% CI 0.33–3.59). The chronotype did not affect daytime sleepiness; the finding of a 2.5-fold increase in risk of poor sleep quality associated with the serotine chronotype (OR = 2.49, 95% CI 0.95–6.56) would also require further testing with the appropriate study size to exclude chance as the determinant. Age, BMI, having minor children, smoking, alcohol intake, and workplace did not affect the risk of either adverse sleep outcome. Replacing the dummy nightshift work variable with a dichotomous variable of the number of night work shifts/month, whether below or above the median of 6, did not show an upward trend by the increasing number of nightshifts per month, leaving substantially unchanged the risk estimates associated with the other covariates (not shown in the Table). There was no interaction between gender and night shift work or sleeping less than 7 h/night; the respective interaction terms did not account for the excess risk associated with the individual covariates and did not improve the fitness of the regression model (not shown in the Table). In the logistic regression model predicting daytime sleepiness, female gender was identified as the only significant contributor (*χ*^2^ = 23.24, *p* < 0.0001), and for poor sleep quality, female gender (*χ*^2^ = 16.09, *p* < 0.0001), sleep deprivation (*χ*^2^ = 25.22, *p* < 0.0001), and the chronotype (*χ*^2^ = 3.96, *p* = 0.047) were significant predictors. The AUC of the model including the whole set of selected covariates was 0.816 for daytime sleepiness and 0.794 for poor sleep quality, suggesting, in both instances, a good quality of the logistic regression models.

## 4. Discussion

According to the U.S. National Sleep Foundation, 44% of women overall report sleep problems [32,33]. Still, data from the 2019 EU Labor Force Survey show that 9.4% of the female workforce works night shifts, with a slightly decreasing trend in the last decade [33]. Additionally, in 2015, 25% of the USA female workforce was involved in shift work, including evening, night, and rotating shifts [34]. Our results confirm that females suffer from daytime sleepiness and poor sleep quality more frequently than males. In our study, 70% of female workers reported sleeping less than 7 h vs. 52% of males; 74% reported poorer sleep quality vs. 40% of males; and 19% reported daytime somnolence vs. 3% of men. Nightshift workers did not sleep fewer hours nor suffered from a poorer sleep quality with reference to daytime workers. Additionally, daytime somnolence was equally prevalent among female nightshift workers and females working daytime. The three men who had an ESS score suggestive of daytime somnolence were nightshift workers, one out of 53 from the ceramic tile factory and two out of 21 hospital workers. The moderate increase in risk of daytime sleepiness in nightshift workers observed in the multivariate analysis was not significant, nor did the risk increase with the number of night shifts per month. The early start of the morning shift in hospitals and factories, by itself, might shorten sleeping time, which, in turn, might account for the elevated baseline prevalence of sleep disturbances in our study. Adding family commitments might further reduce the sleeping hours, as suggested by the elevated risk of daytime somnolence associated with being married. In our study population, nightshift work was not a predictor of daytime sleepiness nor impairment in sleep quality. This would suggest that a regular, forward rotating shift work schedule might effectively promote adaptation to the changes of the sleep–wake cycle. Alternatively, the elevated frequency of sleep disturbances among daytime workers and the small study size might have prevented detecting the role of nightshift work, particularly among female workers. Female shift workers more frequently report taking sleeping pills, more job stress, and more emotional problems [35], as well as shorter and impaired sleep [36], a higher incidence of insomnia [37], and significantly higher sleepiness scores [38,39].

Our results confirm those of a Swedish report that observed an excess of daytime sleepiness and poor sleep quality among female healthcare workers, including 19% shift workers, compared to non-healthcare workers, only 5% of whom were shift workers during the first wave of the COVID-19 pandemic [40]. In another Spanish report, also conducted during the COVID-19 pandemic, female gender, nightshift work, and healthcare work were strong predictors of insomnia, para-insomnia, and an Insomnia Severity Index ≥8 [41]. A review on the prevalence of mental health issues and insomnia among healthcare workers during the first wave of the COVID-19 outbreak further confirmed that female gender and healthcare occupations were the most relevant risk factors [42]. The Swedish authors suggested taking short naps during work breaks, having time-off periods sufficient to recover sleep, and chronotype-aligned work schedules to reduce the excessive daytime sleepiness [40]. We observed the same excess risk among the female workforce independent on the workplace and the COVID-19 pandemic, which, we suspect, might have added to a background difficulty in the adaptation to night shift work.

Gender-related differences in the circadian system might influence nightshift work tolerance and adaptation: for instance, nightshift work is related to disruption of the menstrual cycle and fertility problems [43], and women tend to have a shorter circadian period [44], with a slightly advanced phase and reduced alertness at nighttime [45]. Moreover, childcare and home care would contribute to increasing sleep deprivation and more even so when working nightshifts [17,24,43,45]. The association we observed with marital status would support the hypothesis.

Characteristics of nightshift work, such as duration in hours, years working nightshifts, number of consecutive night shifts, number of night shifts per month, and type of shift rotation scheme, are known to result in circadian disruption, sleep deprivation, and daytime somnolence [46]. In our study, the number of annual nightshifts was lower than the threshold for entitling our study subjects from two workplaces to the benefits awarded to nightshift workers. The limited number of night shifts and the regular forward rotation scheme applied in both workplaces might have allowed a longer recovery time, thus preventing the adverse outcomes of shift work from showing up more frequently in our study population.

We observed an increase in the risk of poor sleep quality associated with the serotine chronotype in respect to the intermediate chronotype. On the other hand, subjects carrying the matutine chronotype were less prevalent among nightshift workers, and there was an inverse association, if any, with risk of daytime sleepiness. Although we cannot exclude chance as a determinant, it is reasonable to postulate that the individual chronotype might address the choice towards a working schedule fitting as much as possible the individual biorhythm. It is also noteworthy that, while we did not observe a difference in the prevalence of specific chronotypes by gender, other studies reported differential age-related changes in males and females, with a tendency of older women to be less morning orientated than older men [47]. A study of Indian police officers showed that poor sleepers improved in terms of daytime sleepiness and sleep quality when their shift work schedule was in phase with their circadian rhythm in respect to the out of phase circumstance [48]. Another study assessed the performances of professional athletes by the time of the day and by their chronotype; the results showed that serotine subjects performed worse early in the morning compared to matutine subjects [49]. Moreover, matutine workers suffered the most in terms of sleep disturbances during nightshifts; the same was observed in serotine workers working early shifts. More studies should address the role of chronotype in facilitating adaptation to nightshift work.

Our study suffers from interpretative limitations due to the small study size and the low prevalence of men with an ESS score ≥11. As a result, risk estimates showed large fluctuations, and the control of confounding factors might have been incomplete. Also, the fact that male participants were twice as many compared to females reduced the statistical power of the analysis, as indicated by the wide confidence interval of the Odds Ratio associated with daytime sleepiness. Still, the advantage of having a small but sizeable proportion of the female workforce also among factory workers provided the opportunity to test the association in two different occupational settings. Therefore, apart from the association of sleep deprivation, daytime sleepiness, and poor sleep quality with the female gender, consistently observed in other studies, we cannot exclude chance as the determinant of our results. As a further limitation, daytime workers in our study might have not been an appropriate reference, as the early start of the first daytime shift in hospitals and factories might be a cause of sleep loss by itself. In fact, daytime workers also slept less than 7 h, the threshold for normal sleep, on average. Moreover, we could not explore other factors known to affect sleep, such as medical condition or socioeconomic status, for instance. However, the socioeconomic status was fairly homogeneous between and within the two cohorts, while information on the medical conditions of study participants was not available. Several medical conditions might have been a reason for unfitness to nightshift work, therefore being over-represented among daytime workers, and they might be associated with poor sleep quality. If so, the resulting bias might have contributed to the lack of a significant association between nightshift work and sleep quality in our study. Additionally, a lifetime history of nightshift work engagement was unavailable. This might have led to underestimating associations if an elevated proportion of daytime workers had previously worked nightshifts. Again, in our study, we relied on self-reported information for outcomes and risk factors, as we did not have information on objective indicators, such as frequency of workplace injuries, body temperature, chronotype, or melatonin and cortisol levels. This is also a drawback further limiting interpretation of our results: we cannot exclude that our observation of a higher prevalence of daytime sleepiness among women, independent of nightshift work engagement, might be due to differential reporting by gender [18,19,21]. Incompleteness of information might be a reason why the results of published reports and metanalyses are so far inconsistent about differential reporting [21,37]. On the other hand, we had the possibility of exploring the mutual effect of multiple variables precluding nightshift work adaptation.

## 5. Conclusions

In conclusion, our results confirm that women are more vulnerable to sleep disruption and experience more difficulty in adapting to nightshift work, even after controlling for marital status, nightshift work, hours of sleep, and chronotype. As recommended by the Working Time Society, a more extensive comprehension of the gender-related differences in adaptation to shift work is needed to design work organization strategies capable of preserving well-being, lowering health risks, and improving workers’ quality of life. While aiming to reduce the risk of workplace incidents and car crashes related to daytime somnolence in commuting back home after a night shift and to improve mental health and well-being among the female workforce such strategies can also promote gender equality in Occupational Safety and Health.

## Figures and Tables

**Table 1:** Selected variables of the study population by workplace and gender.

	Ceramic Tile Workers (N = 91)				Hospital Staff (N = 65)				Total (N = 156)			
	Women		Men		Women		Men		Women		Men	
Age (mean, SD)	44.2	(7.63)	45.1	(8.43)	41.3	(11.4) *	34.2	(8.26) *	42.0	(10.6)	42.7	(8.28)
BMI (mean, SD)	22.3	(2.16) *	25.9	(3.69) *	23.2	(4.65)	23.6	(3.08)	23.0	(4.17) *	25.4	(3.72) *
Total (N, %)	14	(100)	77	(100)	43	(100)	22	(100)	57	(100)	99	(100)
Married (N, %)	11	(78.6) *	52	(67.5) *	17	(39.5) *	6	(27.3) *	28	(49.1)	58	(58.6)
Minor children (N, %)	7	(50.0) *	35	(45.5) *	8	(18.6) *	2	(9.1) *	15	(26.3)	37	(37.4)
Current smokers (N, %)	3	(21.4)	24	(31.2)	4	(9.3)	7	(31.8)	7	(12.3) *	31	(31.3) *
Alcohol intake (N, %)	6	(42.9)	56	(72.7) *	21	(48.8)	16	(72.7) *	27	(47.4) *	72	(72.7) *
Daily	1	(7.14) *	37	(48.1) *	0	(-)	0	(-)	1	(1.75)	37	(37.4)
Occasional	5	(35.7)	19	(24.7)	21	(48.8)	16	(72.7)	26	(45.6)	35	(35.4)
Chronotype (N, %)												
intermediate	5	(35.7)	44	(57.2)	26	(60.5)	12	(54.5)	31	(54.4)	56	(56.6)
matutine	3	(21.4)	23	(29.9)	17	(39.5)	6	(27.3)	20	(35.1)	29	(29.3)
serotine	2	(14.3)	21	(27.3)	9	(20.9)	6	(27.3)	11	(19.3)	27	(27.3)
Sleep deprived (N, %)	9	(64.3)	39	(50.6)	31	(72.1)	13	(59.1)	40	(70.2)*	52	(52.5) *
Work shift (N, %)												
Rotating nightshift	10	(71.4)	53	(68.8)	30	(69.8)*	21	(95.5)*	40	(70.2)	74	(74.7)
Fixed daytime shift	4	(28.6)	24	(31.2)	13	(30.2)	1	(4.5)	17	(29.8)	25	(25.3)
ESS ≥ 11	6	(42.9)	1	(1.3)	5	(11.6)	2	(9.1)	11	(19.3)	3	(3.0)
PSQI ≥ 5	10	(71.4)	21	(27.3)	15	(34.9)	6	(27.3)	25	(43.9)	27	(27.3)

Note. * *p* < 0.05.

**Table 2:** Selected variables of the study population by workplace and by type of work schedule.

	Ceramic Tile Workers (N = 91)				Hospital Staff (N = 65)				Total (N = 156)			
	Daytime		Nightshift		Daytime		Nightshift		Daytime		Nightshift	
Age (mean, SD)	42.2	(8.44)	46.2	(8.45)	47.0	(7.50) *	36.7	(10.8) *	43.8	(8.37)	41.9	(9.13)
BMI (mean, SD)	24.2	(2.68) *	25.8	(2.76) *	24.0	(4.25)	23.1	(4.19)	24.2	(3.24)	24.6	(3.21)
Total (N, %)	28	(100)	63	(100)	14	(100)	51	(100)	42	(100)	114	(100)
Female gender (N, %)	4	(14.3)	10	(15.9)	13	(92.9) *	30	(46.2) *	17	(40.5)	40	(35.1)
Married (N, %)	16	(57.1)	47	(74.6)	4	(28.6)	19	(37.3)	20	(47.6)	66	(57.9)
Minor children (N, %)	11	(39.3)	31	(49.2)	3	(21.4)	7	(13.7)	14	(33.3)	38	(33.3)
Current smokers (N, %)	8	(28.6)	19	(30.2)	1	(7.1) *	10	(19.6) *	9	(21.4)	29	(25.4)
Alcohol intake (N, %)	25	(89.3) *	37	(58.7) *	5	(48.8) *	32	(62.7) *	30	(71.4)	69	(60.5)
Daily	14	(50.0)	24	(38.1)	0	(-)	0	(-)	14	(33.3)	24	(21.1)
Occasional	11	(39.3)	13	(20.6)	5	(48.8)	32	(62.7)	16	(38.1)	45	(39.5)
Chronotype (N, %)												
intermediate	10	(35.7)	32	(50.8)	5	(35.7)	22	(43.1)	15	(35.7)	54	(47.4)
matutine	12	(42.9) *	14	(22.2) *	5	(35.7)	18	(35.3)	17	(40.5) *	32	(28.1) *
serotine	6	(21.4)	17	(27.0)	4	(28.6)	11	(21.6)	10	(23.8)	28	(24.6)
Sleep deprived (N, %)	16	(57.1)	32	(50.8)	13	(92.9)	31	(60.8)	29	(69.0)	63	(55.3)

Note. * *p* < 0.05.

**Table 3:** Results of the logistic regression model predicting an ESS score ≥ 11 and a PSQI score ≥ 6.

	ESS ≥ 11		PSQI ≥ 5	
Covariates	N	OR (95% CI)	N	OR (95% CI)
Age (one year increase)	-	0.99 (0.93–1.04)	-	1.00 (0.96–1.05)
Female gender (vs. male)	17/3	15.9 (4.20–60.0)	42/40	3.91 (1.75–8.73)
Being married (vs. unmarried)	13/3	2.62 (0.78–8.83)	46/36	1.37 (0.54–3.49)
Chronotype				
Matutine (vs. intermediate)	5/7	0.54 (0.14–2.13)	25/22	1.21 (0.49–2.96)
Serotine (vs. intermediate)	8/7	1.09 (0.30–3.92)	35/22	2.49 (0.95–6.56)
Sleep deprivation (<7 vs. ≥7 h)	14/6	1.08 (0.33–3.59)	66/16	5.45 (2.49–11.9)
Night shifts (vs. daytime)	16/4	1.49 (0.33–3.59)	59/23	1.11 (0.46–2.68)

## Data Availability

The data presented in this study are available on request from the corresponding author. The data are not publicly available due to ethical restrictions.
